# Determination of the pK_a_ of the N-terminal amino group of ubiquitin by NMR

**DOI:** 10.1038/srep43748

**Published:** 2017-03-02

**Authors:** Alain Oregioni, Benjamin Stieglitz, Geoffrey Kelly, Katrin Rittinger, Tom Frenkiel

**Affiliations:** 1 MRC Biomedical NMR Centre, The Francis Crick Institute, 1 Midland Road, London NW1 1AT, UK; 2 Molecular Structure of Cell Signalling Laboratory, The Francis Crick Institute, 1 Midland Road, London NW1 1AT, UK

## Abstract

Ubiquitination regulates nearly every aspect of cellular life. It is catalysed by a cascade of three enzymes and results in the attachment of the C-terminal carboxylate of ubiquitin to a lysine side chain in the protein substrate. Chain extension occurs via addition of subsequent ubiquitin molecules to either one of the seven lysine residues of ubiquitin, or via its N-terminal α-amino group to build linear ubiquitin chains. The pK_a_ of lysine side chains is around 10.5 and hence E3 ligases require a mechanism to deprotonate the amino group at physiological pH to produce an effective nucleophile. In contrast, the pK_a_ of N-terminal α-amino groups of proteins can vary significantly, with reported values between 6.8 and 9.1, raising the possibility that linear chain synthesis may not require a general base. In this study we use NMR spectroscopy to determine the pK_a_ for the N-terminal α-amino group of methionine1 of ubiquitin for the first time. We show that it is 9.14, one of the highest pK_a_ values ever reported for this amino group, providing a rational for the observed need for a general base in the E3 ligase HOIP, which synthesizes linear ubiquitin chains.

The modification of proteins with polyubiquitin chains has profound consequences for their behaviour and can target proteins to the proteasome for degradation, activate signalling cascades or regulate the DNA damage response amongst many other functions. The fate of the modified protein depends on the linkage type between ubiquitin molecules within the chain: the C-terminal carboxylate of one ubiquitin molecule can form an isopeptide bond with one of the seven lysine residues within ubiquitin thereby creating seven distinct monotypic chains or, alternatively, form a peptide bond with the N-terminal α-amino group of methionine1 to produce so-called linear or Met1-linked polyubiquitin chains^1^,^2^. These linear chains play important roles in the regulation of immune and inflammatory signalling pathways and contribute to the regulation of apoptotic signalling processes^3^,^4^. They are synthesized by the multi-component E3 ligase LUBAC that consists of three subunits termed HOIP, HOIL-1L and SHARPIN^5^,^6^,^7^. HOIP provides LUBAC with the ability to produce linear polyubiquitin chains in a highly specific manner using its C-terminally located RBR (RING between RING) domain^8^,^9^,^10^. RBR domain-containing E3 ubiquitin ligases form a subfamily of E3s that adopt a hybrid mechanism integrating the properties of RING and HECT-type ligases: a canonical RING domain (“RING1”) initially recognizes the E2~ubiquitin conjugate and subsequently the ubiquitin is transferred onto a conserved cysteine residue located in the RING2 domain of the RBR to form a thioester intermediate before the final transfer of ubiquitin onto a substrate^11^,^12^,^13^. This mechanism is similar to that of HECT-type ligases, which also form a thioester intermediate with ubiquitin, whereas RING-type E3s play a more indirect role and act as a platform to bring the E2~Ub and substrate into close proximity and stabilise a conformation of the E2~Ub conjugate that is primed for ubiquitin transfer^14^. Regardless of the type of E3 ligase involved, for the transfer of ubiquitin onto the substrate to proceed efficiently the incoming nucleophile, which is either the ε-amino group of a lysine side chain or the N-terminal α-amino group of Met1, needs to be in its deprotonated form. The pK_a_ of lysine side chains is around 10.5, meaning they are protonated at physiological pH and hence to be effective nucleophiles a mechanism is required to depress the pK_a_. Acidic residues in E2s, such as Asp127 in the SUMO-specific E2 Ubc9 and Asp117 in UbcH5, have been suggested to contribute to pK_a_ depression during ubiquitin transfer by RING ligases^15^,^16^,^17^,^18^. Similarly, an Asp residue in the active site of the HECT ligase Rsp5 has been reported to play a role in deprotonating the substrate lysine^19^.

In contrast to lysine side chains, the pK_a_ values of the N-terminal amino group in proteins are less well characterised. Values between 6.8 and 9.1 have been reported, with an average of 7.7 ± 0.5^20^. These relatively low values raise the possibility that the synthesis of linear polyubiquitin chains may not require a general base to activate the nucleophile. However, our structural work on the catalytic core of the RBR ligase HOIP – which is the only E3 ligase capable of synthesizing linear ubiquitin chains^8^ - provided a molecular explanation for the observed high chain linkage specificity of HOIP, and highlighted a histidine residue in the active site, His887, that was ideally positioned to carry out the role of a general base^21^. Indeed, substitution of this histidine residue with alanine severely suppresses catalytic activity, which however could be rescued at high pH, strongly indicating that His887 acts as a general base to deprotonate the α-amino group of Met1. This apparent need for a general base prompted us to ask what the pK_a_ value of the N-terminal α-amino group of Met1 might be. Although ubiquitin is one of the best studied proteins and the pK_a_ of most of its ionisable groups have been determined experimentally^22^ we could not find any reports describing Met1 pK_a_ determination. A number of computational approaches exist to calculate pK_a_ values based on the three-dimensional structure of proteins^23^,^24^. However, an overlap of different NMR and crystal structures of ubiquitin showed slight differences in the environment of Met1 and we therefore decided to use an NMR-based approach to determine the pK_a_ experimentally.

NMR chemical shifts are exquisitely sensitive to local ionisation events and serve as excellent reporters in pH titrations. As a result, NMR titrations have been very widely used to measure pK_a_ values in a large range of molecules, including the biologically important ionization of the side-chains of aspartic and glutamic acid and histidine residues of proteins, for which many hundreds of measurements have been reported^20^. In contrast, the literature contains relatively few pK_a_ measurements of N-terminal amino groups. Direct detection of the ^15^N resonance has been used in a small number of studies, but suffers from poor sensitivity^25^,^26^. Higher sensitivity can in principle be achieved by exploiting indirect proton-detected methods such as HSQC and SOFAST, but these methods depend on scalar coupling between the ^15^N and ^1^H nuclei, and are therefore not applicable to free amino groups in aqueous solution where the amino protons exchange with solvent at a rate that is much faster than the scalar coupling.

Alternative strategies involve detection of resonances from nuclei that are more remote from the ionization site, and whilst this approach provides opportunities for using more sensitive NMR methods it creates the risk that the chosen reporters will be affected by ionization events from multiple sites in the protein, resulting in chemical shifts that show complex pH dependence, with concomitant difficulties in interpretation of the data and the possibility of substantial errors in derived pK_a_ values^27^. It is generally recognised that the best reporter is the ionization site itself, where the frequency change associated with the ionization is likely to be largest, followed by the nucleus or nuclei that are covalently bound to the ionization site and other closely adjacent nuclei.

In view of these considerations we have adopted an indirect-detection approach to determine the pK_a_ of the α-amino group of Met1 by monitoring the ^15^N resonance frequency, detected indirectly via the non-labile alpha hydrogen of the terminal residue. This approach is based on that used by André *et al*. to determine the pK_a_ values of side-chain ionizable groups of lysine and arginine residues^28^, and by Lorieau *et al*. for the amino group of the influenza hemagglutinin fusion peptide^29^.

## Results and Discussion

### Data acquisition and analysis

The terminal amino ^15^N data were collected using a two-dimensional version of the HACAN pulse sequence^30^, essentially in the form of Kanelis *et al*.^31^, modified to provide discrimination against the unwanted signals from backbone amide groups and side-chain amino groups of lysine residues. The modifications (described in Methods) reduced the unwanted signals to undetectable levels, and, as a consequence, the assignment of the signal from the N-terminal amino group was self-evident.

The pH titration was carried out by progressive transfer of aliquots between a pair of samples that were initially adjusted to pH 5.8 and 10.5 respectively, as described in Methods. At each stage of the titration the pH of the samples was measured via the ^1^H chemical shifts of a set of indicator molecules present as co-solutes^32^; this avoids the difficulties that arise with conventional electrode-based pH measurement of small-volume samples. The experimental methodology and the resulting pK_a_ values at 21.5 °C for the indicator molecules are described in Methods.

**Figure 1:**
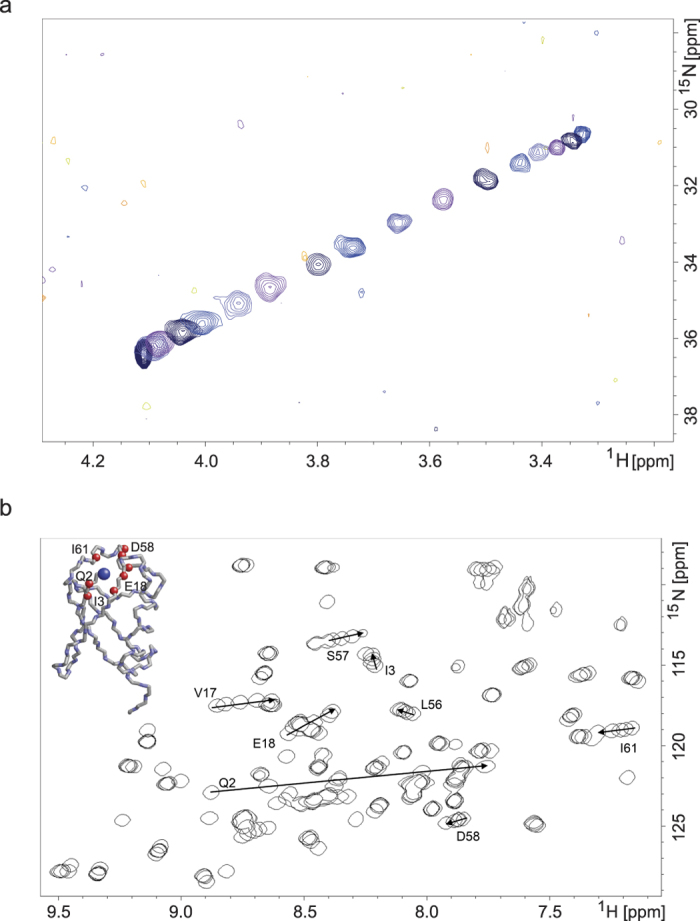
Overlap of ubiquitin spectra at different pH values. (**a**) Overlay of successive 2D HA(CA)N spectra from the titration of human ubiquitin over the pH range 5.8 (lower left) to 10.5 (upper right). The peak arises from the correlation of the terminal ^15^N with the H_α_ of the same residue; the pulse sequence has been adapted so that only this correlation has detectable intensity. In the interests of clarity only a subset of the recorded spectra have been shown on this plot. (**b**) Overlay of ^1^H-^15^N SOFAST-HMQC spectra at the end-points of the titration and three intermediate pH values. The peaks that show appreciable pH dependence in this range are annotated; the arrows show the movement of the peaks with increasing pH. In the inset structural diagram (1UBQ.pdb)^50^ the backbone nitrogen atoms corresponding to these peaks are shown as red spheres, and the N-terminal amino group is shown in blue).

The results of the HA(CA)N experiment are shown in Fig. 1a, and although the primary purpose of this experiment was to detect the ^15^N chemical shift of the terminal nitrogen, the alpha hydrogen clearly also shows a pH dependence that is strongly correlated to that of the ^15^N. In addition to the HA(CA)N experiment, a two-dimensional ^1^H-^15^N SOFAST-HMQC data set^33^ was recorded at each pH point to assess the integrity of the protein, which was found to remain folded up to the highest pH used in this work (Fig. 1b). In the SOFAST spectra a number of cross-peaks migrate as a function of pH, most notably the peak from the backbone amide of Gln2, and the coordinates of this peak were also analysed.

**Figure 2:**
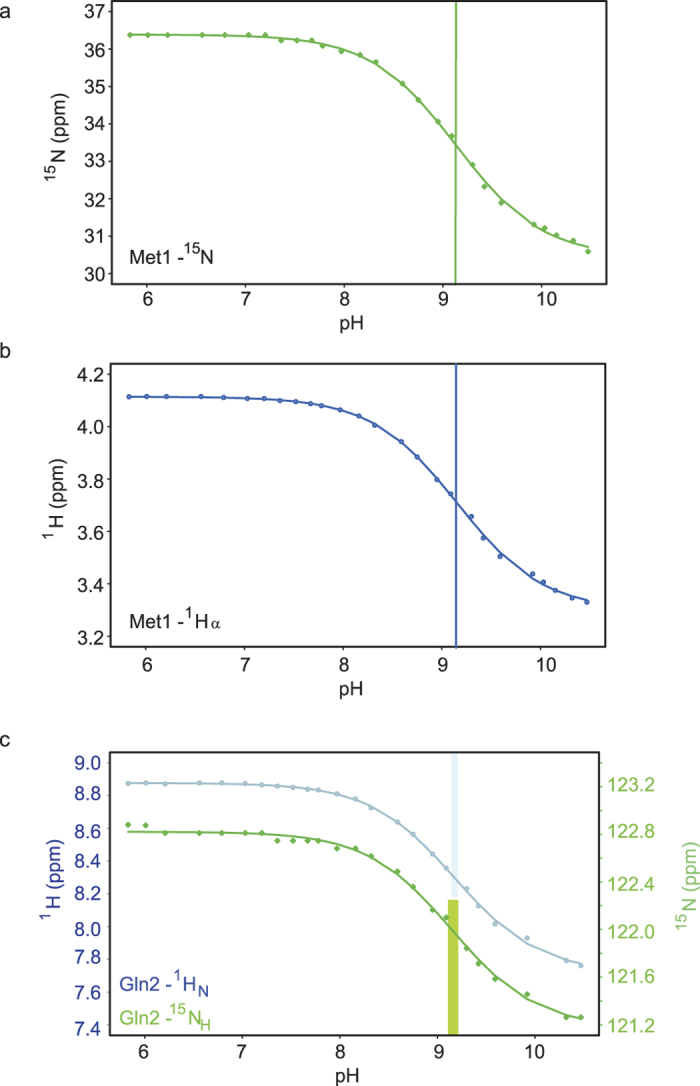
Chemical shift dependence on pH. The chemical shift dependence on pH of (**a**) Met1-^15^N and (**b**) Met1- ^1^H_α_ measured with the HA(CA)N experiment; (**c**) Gln2-^15^N and H_N_ measured with ^1^H-^15^N SOFAST-HMQC. The solid lines are the curves fitted to the Henderson-Hasselbalch equation. The widths of the vertical bars denote the 95.45% confidence intervals of the fits, as determined by the statistical bootstrap.

The peak positions for the ubiquitin titrations were fitted using non-linear regression to the Henderson–Hasselbalch equation, recast in terms of chemical shifts:


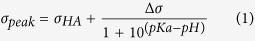


where σ_peak_ is the measured NMR chemical shift of the peak of interest, σ_HA_ is the chemical shift of the protonated form and Δσ is the difference between σ_HA_ and the shift of the deprotonated form. All fitting and statistical analysis was carried out using the statistics package R, as described in Methods^34^. Good fits to eq. (1) were obtained for all four measured ubiquitin chemical shifts (Fig. 2).

### pK_a_ of the N-terminal amino group

The pK_a_ values derived from the NMR measurements carried out on the four nuclei at or near the N-terminal residue are in close agreement (Table 1). The pK_a_ derived from the ^15^N chemical shift of the terminal amino group is 9.14; this nucleus is reasonably assumed to be the most faithful reporter. The values from the other three measured nuclei fall in the range 9.15 to 9.18, with fitting confidence intervals which overlap that of the Met1-N determination. This suggests that all of the observed chemical shift changes reflect the N-terminal amino group ionisation event alone. This is unsurprising as the measured value lies in a pH window devoid of other typical ionisations in proteins. The absence of other ionization events in this pH range is further evidenced by an examination of the full set of peaks in the SOFAST ^1^H-^15^N-correlation spectrum, where all of the ^1^H-^15^N correlations which display appreciable titration behaviour in this range arise from atoms that are in close proximity to the N-terminus in the three-dimensional structure (Fig. 1b).

## Conclusions

**Figure 3:**
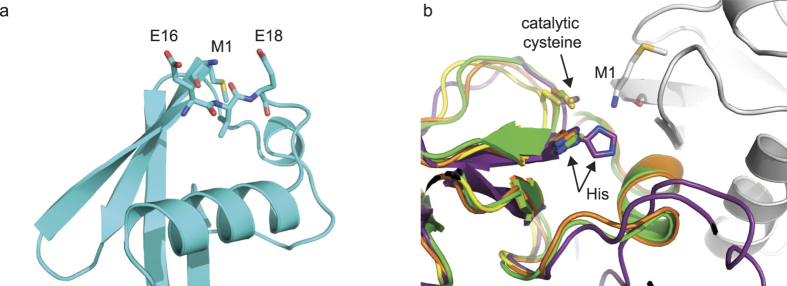
Structural environment of Met1 of ubiquitin and active site of RBR ligases. (**a**) Structure of ubiquitin highlighting the residues close to Met1 (1UBQ.pdb)^50^. (**b**) Overlap of the structures of HOIP in purple (4LJO.pdb)^21^, Parkin in green (5CAW.pdb)^40^ and orange (5C23.pdb)^38^ and HHARI in yellow (4KBL.pdb)^36^ zoomed in onto the active site. The catalytic Cys885 and His887 of HOIP, plus Met1 of the acceptor ubiquitin (in gray) are shown.

The pK_a_ of 9.14 for the N-terminal amino group of ubiquitin, determined from the ^15^N chemical shift, is at the upper limit of experimental values for proteins reported in the literature, which span 6.8 to 9.1, and is also higher than that determined for model peptides^20^,^35^. The α-amino group of Met1 is solvent exposed making it difficult to rationalize why its pK_a_ is higher than in most other cases and we speculate that the proximity of Glu16 and Glu18 (Fig. 3a) create a negatively charged environment that raises the pK_a_. Interestingly, the conserved histidine residue in HOIP that we previously proposed to act as a general base to deprotonate the N-terminal amino group is conserved in a number of RBR ligases, all of which transfer ubiquitin onto a lysine sidechain^21^. At present the structure of the HOIP/ubiquitin complex is the only snapshot of an RBR ligase/substrate active site. The only other structures of RBR ligases available are of HOIP bound to E2~ubiquitin, and HHARI and Parkin in the autoinhibited state, or partially active forms of Parkin^36^,^37^,^38^,^39^,^40^,^41^. The histidine is in a different conformation in those structures (Fig. 3b) and points away from the ubiquitin which could indicate that substrate binding might either induce changes around the active site in HHARI or Parkin or that the substrate is presented to the active site in a different orientation in these RBRs. Further studies are required to fully understand how the incoming nucleophile is activated in other RBRs.

## Methods

### Internal pH indicator validation

We used the set of internal indicator molecules proposed by Baryshnikova *et al*.^32^ – Tris, formate, piperazine, and imidazole - but first re-determined the indicator pK_a_ and limiting chemical shift values at 21.5 °C, the temperature of interest in this study. We also re-assessed the range of applicability of each indicator for this temperature, and extended the applicability to a higher pH range by utilising the second ionisation of piperazine. The chemical shifts of the titrating signals of Tris, formate, and imidazole-H2 were analyzed using the Henderson-Hasselbalch equation (in the form shown above as eq. (1)), whereas the piperazine chemical shift was fitted to the extended form of the equation which accommodates two ionization events^42^:


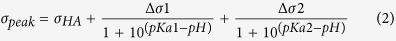


where Δσ1 is the variation of chemical shift associated with the titration of the deprotonation governed by *pK*_*a*_*1* and Δσ2 the added chemical shift variation governed by *pK_a_2*.

The sample used for these measurements consisted of a solution of 100 mM KCl, 2 mM Tris, 2 mM formate, 2 mM piperazine, 2 mM imidazole and 0.2 mM DSS in 95% H_2_O-5% D_2_O, matching the buffer used subsequently in the ubiquitin measurements. The pH was adjusted across the desired range and measured using a glass pH electrode.

**Figure 4:**
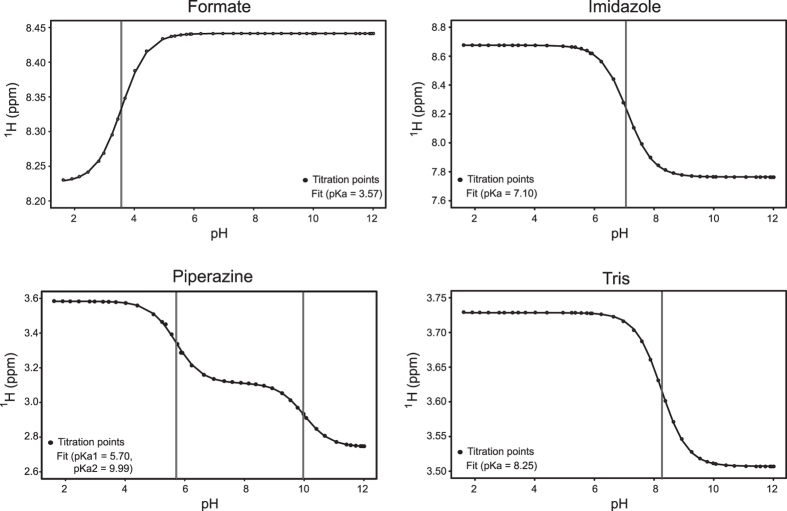
Titration curves of internal NMR reporters. pH titration curves for the four internal NMR reporters: formate, piperazine, imidazole-H2 and Tris. The solid lines are the curves fitted to the Henderson-Hasselbalch equation as described in the text. The resulting pK_a_ values are marked with vertical lines.

The resulting limiting chemical shifts and pK_a_ values (Fig. 4 and Table 2) agree well with those reported by Baryshnikova *et al*. at 30 °C, demonstrating the validity of their observation that this approach is quite robust with respect to changes in temperature^32^.

### NMR sample preparation

Recombinant wild-type human ubiquitin was expressed as untagged protein using pET15 and *E. coli* strain BL21(DE3), and purified by anion exchange using a Q Sepharose resin followed by gel filtration on a Sephadex G-100 column (GE Health-care). ^13^C/^15^N labelled ubiquitin was obtained by expression in minimal medium containing glucose-^13^C_6_ and ^15^NH_4_Cl as sole carbon and nitrogen sources.

Two 0.5 mM ubiquitin samples for NMR were prepared by dialysis into a 95% H_2_O-5% D_2_O buffer (100 mM KCl, 2 mM Tris, 2 mM formate, 2 mM piperazine, 2 mM imidazole, 0.2 mM DSS). The two initial samples were adjusted to pH 5.8 or pH 10.5. Intermediate pH values were attained by transferring small aliquots between samples after each set of NMR measurements. The sample that was initially at pH 5.8 thereby increased with each transfer, whilst the pH of the other sample decreased. NMR measurements were made on both samples. Accurate sample pH was monitored after each transfer using the ^1^H chemical shift of the appropriate buffer component as an internal indicator, making use of values for pK_a_, σ_HA_, σ_A_ and applicability regions re-derived experimentally for 21.5 °C as shown in Table 2.

### NMR measurements

All NMR measurements were performed on a Bruker Avance 600 MHz equipped with a 5 mm TCI cryoprobe, at a sample temperature of 21.5 °C, as demonstrated by a prior calibration using the method of Findeisen *et al*.^43^.

Three NMR spectra were acquired at each pH value: a 1D ^1^H spectrum using excitation sculpting^44^, a 2D ^1^H-^15^N SOFAST-HMQC spectrum^33^, and a 2D HA(CA)N spectrum^30^,^31^. The latter was based on the sequence of Kanelis *et al*.^31^, adapted to confer selectivity and improve sensitivity for the desired N-terminal amino signal. Selectivity with respect to backbone amide signals was achieved by replacing the rectangular ^15^N inversion pulses of the standard sequence with Q3-shaped selective pulses^45^, centred at a ^15^N offset of 35 ppm, and having a duration of 1.3 ms. Discrimination against signals from the side-chain amino groups of lysine residues was obtained by setting the delay within the sequence that serves for evolution of proton-coupled ^13^C magnetization to 3.4 ms, a value that was optimal for the methine Cα group of N-terminal methionine and simultaneously minimized the signal from the methylene Cε groups of lysine side-chains. 2D spectra were processed using nmrPipe^46^. ^1^H chemical shifts were referenced to internal DSS; ^13^C and ^15^N shifts were referenced indirectly using the gyromagnetic ratios of Wishart *et al*.^47^. Chemical shifts for the signals used in the ubiquitin pK_a_ analysis are given in Table 3.

### Statistical methods

Statistical analysis and Henderson-Hasselbalch equation fitting were done using R^34^. Both the NMR reporters and the ubiquitin titration curves were fitted using a non-linear least-squares method (Gauss-Newton). Because of the non-linear nature of the Henderson-Hasselbalch equation, the pK_a_ values here are given with estimated confidence intervals rather than standard deviations^48^. Confidence intervals of 95.45% were calculated using a bootstrap procedure^49^; this interval corresponds to ± 2σ for a normal distribution. The bootstrap calculation was run with N = 1000 and incorporated the bias correction and acceleration methods, which improve the accuracy by taking into account the non-normality of the bootstrap distribution^48^.

## Additional Information

**How to cite this article:** Oregioni, A. *et al*. Determination of the pK_a_ of the N-terminal amino group of ubiquitin by NMR. *Sci. Rep.* **7**, 43748; doi: 10.1038/srep43748 (2017).

**Publisher's note:** Springer Nature remains neutral with regard to jurisdictional claims in published maps and institutional affiliations.

## Figures and Tables

**Table 1:** pK_a_ values derived from chemical shift measurements.

Measured nucleus	NMR experiment	pK_a_	CL−	CL+	σ_HA_	σ_A_
Met1-^15^N	HA(CA)N	9.14	9.105	9.163	36.386	30.444
Met1-^1^H_α_	HA(CA)N	9.16	9.133	9.188	4.114	3.301
Gln2-^1^H_N_	^1^H-^15^N SOFAST-HMQC	9.18	9.143	9.209	8.877	7.718
Gln2-^15^N	^1^H-^15^N SOFAST-HMQC	9.15	9.101	9.203	122.821	121.178

pK_a_ values derived from chemical shift measurements of four nuclei (Met1-^15^N, Met1-^1^H_α_, Gln2-^1^H_N_ and Gln2-^15^N) from two NMR sequences (HA(CA)N and ^1^H-^15^N SOFAST-HMQC). CL− and CL+ are the lower and upper limits of the fitting confidence intervals calculated at the 95.45% confidence level as described in the text. The limiting chemical shifts (in ppm) obtained from the non-linear regression analysis are denoted by σ_HA_ and σ_A_ for the protonated and deprotonated forms respectively.

**Table 2:** Measured pK_a_ values of four internal NMR reporters.

	pK_a_	σ_HA_	σ_A_	Lower pH limit	Higher pH limit
Formate	3.57	8.227	8.442	2.6	4.6
Piperazine pK_a_1	5.70	3.584	3.111	4.7	6.7
Imidazole	7.10	8.675	7.765	6.1	8.1
Tris	8.25	3.729	3.507	7.2	9.2
Piperazine pK_a_2	9.99	3.111	2.744	9.0	11.0

Measured pK_a_ of the four internal NMR reporters, where σ_HA_ and σ_A_ are the chemical shifts (in ppm) of the protonated and deprotonated state respectively. The second pK_a_ of piperazine has been included to extend the range of measureable pH. The suggested range of applicability (limits calculated as pK_a_ ± 1) of each reporter is also shown.

**Table 3:** Ubiquitin titration data.

pH	Met1-^1^H_α_	Met1-^15^N	Gln2-^1^H_N_	Gln2-^15^N
5.83	4.114	36.37	8.874	122.88
6.01	4.115	36.37	8.877	122.87
6.21	4.115	36.37	8.871	122.81
6.56	4.115	36.37	8.877	122.81
6.79	4.111	36.37	8.877	122.81
7.03	4.107	36.37	8.873	122.81
7.2	4.107	36.37	8.865	122.81
7.36	4.099	36.23	8.858	122.75
7.52	4.095	36.23	8.850	122.75
7.67	4.088	36.23	8.838	122.75
7.78	4.080	36.08	8.834	122.75
7.97	4.064	35.94	8.811	122.68
8.16	4.041	35.84	8.779	122.68
8.32	4.005	35.65	8.724	122.62
8.59	3.943	35.08	8.638	122.49
8.75	3.884	34.64	8.564	122.36
8.95	3.798	34.06	8.443	122.17
9.09	3.743	33.67	8.357	122.10
9.3	3.657	32.90	8.231	121.84
9.42	3.575	32.33	8.126	121.71
9.59	3.505	31.89	8.016	121.59
9.92	3.438	31.31	7.930	121.46
10.03	3.407	31.22	N.D.	N.D
10.15	3.375	31.03	N.D.	N.D.
10.32	3.346	30.88	7.793	121.26
10.47	3.330	30.59	7.762	121.26

Chemical shifts (in ppm) for the ubiquitin signals used in the Met1 pKa determination, measured from HA(CA)N (Met1) and SOFAST-HMQC (Gln2) recorded at the indicated pH values. N.D. – not determined owing to peak overlap.
